# QT prolongation with methadone

**DOI:** 10.4103/0972-5229.40954

**Published:** 2008

**Authors:** Radhika Dhamija, Susan Bannon

**Affiliations:** Department of Medicine and Pediatrics, KCMS, Michigan State University, Kalamazoo, MI, USA; 1 Department of Medicine, KCMS, Michigan State University, Kalamazoo, MI, USA

Sir,

Prolonged QT interval can lead to Torsades de pointes and is an established risk factor for sudden death. Though prolonged QT-interval has been conventionally linked to many antiarrhythmic and antipsychotics drugs,[1] it is being increasingly recognized in relation to opiod agonist like methadone.[2–5]

A 42-year-old Caucasian male, came to the Emergency Room in a disoriented, confused and drowsy state following inadvertent consumption of “his friend's pain control pills” which he had taken for his chronic back pain relief. On further history it was found that he had consumed methadone pills (at least three). The pills were not his own prescription medication. On physical examination his temperature was 98.8 °F, respiratory rate 20/min and his pupils pin point. His EKG showed QTc interval of 520 msec, heart rate of 113/ min. His serum studies showed potassium of 4.2 mmol/l, sodium of 136 mmol/l and calcium of 2.15 mmol/l. His urine drug screen was negative. There was no known history of arrhythmias in the family nor did the patient have any history of palpitations. There was no history of any other drug intake. His past medical history was significant for only chronic back pain, while his past surgical history was non-contributory.

He was given Naloxone 0.4 mg as antidote in the ER. A repeat EKG after 12h showed heart rate of 81/min and QTc interval of 450 msec.

Methadone is a long-acting synthetic opiod (opiod), pharmacologically very similar to morphine, used in the management of acute and chronic pain syndromes and opiod de-addiction. The methadone metabolite levacetylmethadol (produced in the liver) is effective in the treatment of opiod dependency, but has been linked with QT prolongation, torsade de pointes and sudden death. For this reason levacetylmethadol was withdrawn from the European market. The half life of methadone is between 8-59 hours, due to high tissue binding and slow release from these tissue proteins. This explains the negative drug screen that can occur as in our patient.[6] The effect on QT interval is dose dependent.[4]

In our patient acute overdose of methadone had led to prolongation of his baseline QTc interval of 450 msec to 520 msec. Methadone is been increasingly used by primary care physicians for pain management. Because of its role in QT prolongation, a baseline EKG is warranted before starting the drug. Patients with a history of narcotic drug abuse on methadone maintenance should be screened for prolonged QT interval by measuring their baseline and periodic EKG's.
